# Fourteen years’ clinical experience and the first million babies protected with human live-attenuated vaccine against rotavirus disease in Italy

**DOI:** 10.1080/21645515.2021.1955611

**Published:** 2021-08-09

**Authors:** Paolo Bonanni, Giorgio Conforti, Elisabetta Franco, Giovanni Gabutti, Federico Marchetti, Antonella Mattei, Rosa Prato, Giovanni Vitali Rosati, Francesco Vitale

**Affiliations:** a Department of Health Sciences, University of Florence, Italy; b Italian Federation of Primary Care Pediatricians (FIMP), Genoa, Italy; c Department of Biomedicine and Prevention, University of Rome Tor Vergata, Rome, Italy; d Department of Medical Sciences, University of Ferrara, Ferrara, Italy; e GSK, Verona, Italy; f Department of Life, Health & Environmental Sciences, University of L’Aquila, L’Aquila, Italy; g Department of Medical and Surgical Sciences, University of Foggia; Department of Hygiene, Policlinico Riuniti University Hospital of Foggia, Foggia, Italy; h Family Pediatrician (FIMP Federazione Italiana Medici Pediatri), Greve in Chianti, FI, Italy; i Department of Health Promotion, Maternal-Child Care, Internal Medicine and Medical Specialties, University of Palermo, Palermo, Italy

**Keywords:** Rotavirus, Italy, vaccination, epidemiology, safety, impact

## Abstract

Rotavirus (RV) causes up to half of hospital and community acute gastroenteritis (AGE) cases in young children in Italy. Two RV vaccines, available since 2006, are human RV (HRV) and human bovine RV (HBRV). This report looks back at the implementation of RV vaccination with HRV in Italy, and at HRV current and future perspectives. Initial regional policies led to national implementation by 2018, after scientific societies’ disease awareness efforts. Following vaccination, RV hospitalizations declined significantly, and cost savings were observed. The two-dose HRV vaccine is easily administered during compulsory vaccine visits, helping increase coverage. Intussusception, a serious event in children <1 year, was reported in Italy with a rate of 33–40 per 100,000 infants. RV vaccination presents a low increased risk of intussusception after the first dose, estimated at 0.6 cases per 100,000 doses in Italy in 2019. Parents should be aware of the intussusception risk and symptoms to ensure prompt treatment. It is widely recognized that the vaccination benefits (large numbers of RV hospitalizations prevented) outweigh the risk. HRV introduction in Italy was supported by epidemiologic burden studies, healthcare provider opinions, and congress debates, which significantly contributed to implementation of RV universal routine infant vaccination in Italy.

## Graphical abstract

**Figure:**
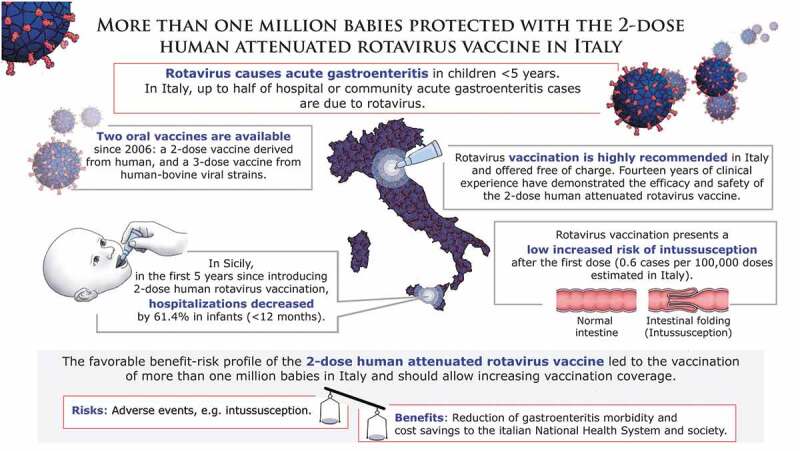

## Introduction

Rotavirus (RV) is the most common cause of gastroenteritis in children under 5 years, with a full range of severity of clinical presentations, which may lead to emergency department visits and hospitalizations, even in industrialized countries. Highly contagious RV gastroenteritis (RVGE) also leads to nosocomial infections.^1^

Since the availability in 2006 of two RV vaccines to protect infants and young children against RVGE, and the 2009 World Health Organization (WHO) recommendation that all countries should implement RV vaccination, the response in countries has been varied. Few European countries, the United States (US) and Australia rapidly adopted RV vaccination while other countries delayed the decision to consider RV universal routine vaccination (URV).^1^

RV vaccination in Italy has evolved. Two RV vaccines were made commercially available since 2006, but only 10,000 babies were vaccinated in Italy in 2009, less than 2% of estimated newborns.^1^ However, supported by the growing international body of evidence and the scientific societies positions, RV URV in Italy was first implemented at the regional level and finally at the national level in 2018. During this timeframe, human live-attenuated RV vaccine (HRV; *Rotarix*, GSK) was introduced into clinical use and followed by a number of publications, which supported healthcare providers in recommending the vaccine. Up to 2020, approximately 1.2 million babies were vaccinated with HRV in Italy (data on file). This report looks back at the implementation of RV vaccination with HRV in Italy and at its current and future perspectives.

## Epidemiology

Before RV vaccination was implemented in Italy, studies estimated that a third to half of acute gastroenteritis (AGE) seen in the hospital was due to RV infection (e.g., 50.8% in the Lombardy region^2^ and 28.6% in the Veneto region^3^), while the incidence of nosocomial RV infection was around 5%.^4^ RV hospitalization incidence was found to be highest in younger children^5^ (e.g., 255 vs 177 per 100,000 for children aged <1 year vs <5 years^6^). Similarly, around a third to half of community AGE cases were due to RV infection (e.g., at peaks, 49.1% in winter 2004–2005 and 53.9% in spring 2005), with the highest incidence in children <2 years old.^7,8^ A family pediatrician reported that the incidence of AGE in his practice was, surprisingly, as high as that of otitis and cough (5%), confirming the RV burden of disease (BoD) for the family pediatrician and the families.^9^

## Design and development of RV vaccines

Mechanisms eliciting protection, following natural RV infection, or live RV vaccination, have not been clearly defined. The two principal hypotheses on protection mechanisms differ on one key point: the role of serotype-specific neutralizing antibody.

Multicomponent vaccines, such as *Rotashield* (Wyeth Lederle Vaccines SA) or reassortant human bovine RV vaccine (HBRV; *RotaTeq*, Merck & Co), have been developed to stimulate neutralizing antibody against all major RV serotypes, based on the idea that protection comes from antibody that recognizes serotype-specific neutralization epitopes.^10^ However, following RV infection, effectors that may elicit protection include non-neutralizing antibodies and T cells.^11^ Therefore, the development of single-component RV vaccines, like *Rotarix* (HRV), was based on the idea that protection is multifactorial, elicited by immune effectors like cytotoxic T lymphocytes other than just neutralizing antibody.^10,11^ Following vaccination, cluster of differentiation 4 (CD4)-bearing T cells may produce antiviral cytokines earlier or in greater quantities than following primary infection; in vitro, RV replication is blocked by several cytokines.^12^ Plotkin et al. (2017)^12^ report several possible mechanisms for heterotypic protection including “antibodies against cross-reactive epitopes on outer capsid proteins VP4 and VP7, antigenically conserved inner capsid proteins that are actively transported through rotavirus-infected villous epithelial cells, rotavirus specific cytotoxic T lymphocytes that broadly cross-react with cells infected with different rotavirus serotypes, or antiviral cytokines generated by activated CD4-bearing T cells.”^12^ Post-marketing surveillance studies have confirmed that HRV induces heterotypic protection, as reported in the label (Summary of Product Characteristics [SmPC] *Rotarix*).^13^

The development of HRV began in Cincinnati in 1988, during a clinical trial of a bovine-derived RV strain that proved to be ineffective.^14^ Serendipitously, in that season in Cincinnati, only one RV serotype, G1P8, was circulating. At the end of the trial, it was observed that natural infection with G1P8 could provide protection, even after asymptomatic infection, and that this strain induced neutralizing antibodies at least for G1–G4 serotypes. Thus, it was decided to use the clinical isolate named 89–12 for a human derived, live-attenuated oral vaccine development.^14^ The strategy to attenuate the wild strain was to passage it multiple times in cell culture, the same technique used by Sabin for the production of the polio oral vaccine. The 89–12 strain was passed 33 times in African Green Monkey kidney cells after which it proved to be safe and efficacious in a two-dose schedule in a double-blind, placebo-controlled, multicenter, randomized efficacy trial enrolling 213 healthy infants.^14^ Since then, the final vaccine formulation, named RIX4414, entered the development phase with large clinical trials in many countries around the world.^14^ Clinical studies from Europe and Latin America showed a favorable benefit-risk profile, clearly demonstrating the vaccine’s efficacy and with no increased risk of intussusception observed.^13,15^

In 2006, the European Medicines Agency approved HRV for the active immunization of infants for prevention of gastroenteritis due to RV infection.^16^ The two-dose oral HRV vaccine can be given from 6 weeks of age, with at least 4 weeks between doses, and the course must be completed by 24 weeks of age. Later that year, a second RV vaccine, HBRV,^17^ was also approved in Europe. Both HRV and HBRV are effective in preventing RV, with good safety profiles, HBRV is administered in a three-dose schedule.^18,19^

## Assessing and building disease awareness in Italy

**Figure 1.**
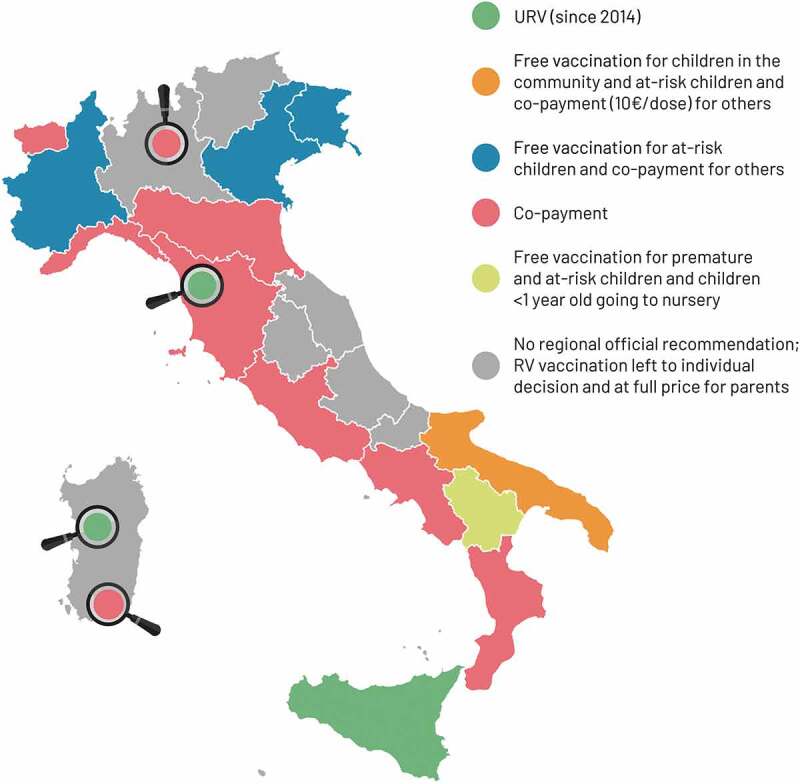
Introduction of rotavirus (RV) vaccination in different regions of Italy (modified with permission from^22^). In 2014, only one Italian region (Sicily) and some health authorities actively offered free RV vaccination. The Puglia region introduced RV vaccination for all newborns at a discounted co-payment of €10.00/dose for families. Other regions offered free vaccination to defined categories of infants.^22^ Magnifiers highlight municipality-specific type of rotavirus vaccination offer. RV, rotavirus; URV, universal routine vaccination.

At first, the RV BoD and value of RV vaccination was under-recognized by pediatricians or parents. Regions in Italy had different vaccination policies in place (Figure 1), and vaccination acceptance and coverage was heavily influenced by whether the vaccines were introduced in the Regional Calendar and if they were offered free of charge and promoted by healthcare providers. A survey carried out in 2009 assessing the views of public health officials found that only 52.4% would recommend adding RV vaccination to the National Immunization Program (NIP) free of charge.^20^

A survey of family pediatricians carried out in 2013 showed that half of the respondents recommended RV vaccination and thought it should be offered to all children for free by the Local Health Unit (*Azienda Sanitaria Locale*, ASL), and 35% thought that the ASL should offer it with co-payment by the family.^21^

A study in 2015, in Italian healthcare providers attending educational courses, found that 57.4% routinely recommended RV vaccination, but most reported that fewer than a quarter of vaccination attendees adhered to RV vaccination, due to skepticism about the vaccine (60.4%) or because cost was a barrier (34.1%). If the vaccine was free, 81.1% of the healthcare providers would recommend it.^23^

A primary care study using a validated quality of life questionnaire reported that acute RVGE caused parents worry and distress and had a negative impact on their daily lives.^24^ The majority (93.6%) of parents who had a child hospitalized for RVGE experienced high/medium stress, 74.5% were not aware of RV vaccination and nearly 80% would, after this experience, strongly recommend it to other parents.^25^ In 2018, in the Naples area, RV vaccination was recommended and provided free of charge, yet only 15.3% of parents surveyed had vaccinated their child, while more than half of the other parents wanted to, but did not, due to lack of knowledge (77.9%) and because it had not been recommended by their family pediatrician (31.6%). Public education programs were found to be needed to increase coverage of RV vaccination.^26^

Despite strong evidence on the vaccine’s efficacy and safety, and on the cost impact in other countries, it was not until 2017 that RV vaccination was included in the NIP and started to be increasingly known and accepted throughout Italy. In 2018 compared with 2017, knowledge about vaccine recommendations in a sample of healthcare providers attending educational courses had increased (95% vs 90%), more participants recommended the vaccine routinely (82% vs 76%), and more participants said >75% of their patients chose to get vaccinated (33% vs 11%). Parents’ acceptance of vaccination was driven by fear of severe gastroenteritis (50%) and by the national recommendation to vaccinate (24%). Parents’ reasons for refusal to vaccinate were skepticism (23% vs 55%), because it was not mandatory (34%), fear of intussusception (21%), and not recommended by a health professional (18%). Overall, RV coverage was increasing and may be due to health professional education, which may have increased recommendations for non-mandatory recommended vaccines.^27^

A second survey carried out in 2017 showed that 96.7% of family pediatricians were aware of the actively recommended and non-obligatory status of RV vaccination. Compared to the 2013 survey,^21^ there was an improvement in scientific knowledge on RV vaccines and a consistent increase (85.8% vs 48.4%) in sharing the opportunity of the free and active offer of RV vaccination.^28^

In 2012, *Calendario della Vita* (CdV), a committee including four scientific societies: Italian Society of Preventive Medicine and Hygiene; Italian Federation of General Practitioners; Italian Society of Pediatrics; and Italian Family Pediatrician Association,^29^ provided vaccine recommendations with supporting evidence. The objective was to help improve regional vaccination policies. In 2012, the CdV recommended RV vaccination with a co-payment by the family to cover part of the vaccine and administration cost.^29^ From 2014, CdV recommended universal RV vaccination free of charge, and advised family pediatricians to help implement it in 2019.^30–32^

Different Italian regions took different approaches to providing access to RV vaccination. Sicily was the first region in 2013 to implement universal RV vaccination actively offered and free of charge.^25^ In February 2017, the Triennial National Vaccination Plan (PNPV 2017–2019)^33^ aimed to overcome regional differences by recommending universal RV vaccination to all children over 6 weeks of age, free of charge, based on the CdV recommendations. The objective was to increase coverage to ≥60% (2017), ≥75% (2018), and ≥95% (2019).^33^ In June 2017, the mandatory vaccination law was introduced. However, RV vaccination was not listed as a mandatory vaccination but was strongly recommended, like meningococcal and pneumococcal vaccines, and included in the Essential Levels of Assistance list (*Livelli Essenziali di Assistenza*).^34^ Regional decision-makers’ knowledge, attitude and beliefs about vaccination were therefore crucial for vaccine implementation.^23^

The Ministry of Health advises that recommended vaccines (such as RV) should be actively promoted through post, e-mail and short message service (SMS), and that vaccination services should ensure adherence to both mandatory and recommended vaccination, through informative interviews during each visit. Family pediatricians play a key role and must get involved in promoting vaccination.^34^

## Impact of regional URV implementation

In the first year after RV vaccination (with 35% coverage in 2013), the mean number of children hospitalized for RVGE in Sicily dropped by 39.3% and 48.3% (in children <5 years and <1 year, respectively).^35^ The number of hospitalizations for intussusception did not change (15 cases in 2013 vs 15.4 per year between 2003 and 2012, for children <1 year).^20^

In the first 5 years of vaccination (with an average coverage across Local Health Units in Sicily of 38.2%), RVGE hospitalization rates continued to decrease in the post- versus pre-vaccination periods; by 61.4% (aged <1 year), by 51.2% (aged 12–23 months), and by 48.8% (aged 24–35 months) with smaller decreases (around 25%) in ages 36–59 months.^36^ The highest coverage (58.6%) was achieved in Trapani, which had a 56.5% reduction in hospitalization, while the lowest coverage (19.1%) was in Messina, which had a 15.7% decrease in hospitalization.^36^

Despite low coverage (peak of 45% in 2016), there was a decline in average RVGE hospitalization costs and numbers of cases hospitalized in Sicily, resulting in cost savings of €1,134,056 per year (for direct medical, non-medical, and indirect costs) following RV vaccination.^37^

## Health economics

Studies found that AGE due to RV was typically more severe than other causes^38–42^ resulting in high costs, for example, €1,536 (interquartile range [IQR] 1,279–1,608) per hospital case in Sicily,^43^ nosocomial case costs of around €8.02 million per year in Italy,^4^ and regional costs of up to €700,000 (Emilia Romagna region),^44^ or over €1 million (Veneto region)^3^ per year (Supplementary Table 1).

Early costing studies of the outpatient costs of AGE in children in Italy found that costs were higher for younger children (mean cost of €116 vs €72 for <36 months vs >36 months) and that around 75% of this cost was due to lost productivity in family members.^45^

Subsequent economic analyses of RV vaccination with HRV confirmed the impact of societal costs and consistently found vaccination to be cost-saving from a societal perspective.^46–49^ In addition, HRV was found to be cost-effective from a health payer’s perspective (i.e., cost per quality-adjusted life-year [QALY] of €14,829), as it prevented a significant number of cases, hospitalizations and medical visits.^47^ When adding the impact of herd immunity provided by RV vaccination, HRV was also found to be cost-saving from a health payer’s perspective. The model predicted vaccination would result in 71% fewer RVGE cases and 86% fewer hospitalizations, with an impact on quality of life and mortality, saving the National Health System over €14 million in costs.^48^ An economic analysis of RV vaccination in Italy estimated that RVGE with no vaccination would cost €48.2 million (€90.8 per patient) with a QALY loss per patient of 0.0006.^49^

## Implementation of the two-dose HRV schedule

The Ministry of Health’s aim was to increase RV vaccination coverage to over 95% by 2019,^33^ acknowledging that the mean national coverage is lower than expected to date, for instance, 26.15% in 2019 (birth cohort 2017) with consistent differences across regions.^50^ However, in the Lombardy region, where RV was actively offered, coverage exceeded the 60% target in 2018 in the first year of URV implementation. The key drivers of this rapid increase in coverage were: a) good collaboration between vaccine services and pediatricians; b) the information interviews clearly discussing the risk-benefit profile; and c) from a practical viewpoint, the ease of administering two-dose HRV with other vaccines given at 3 and 5 months of age.^51^

Studies from Italy and other countries have shown good coverage, compliance and adherence to vaccination with the two-dose HRV vaccine.^19^ Two years after universal RV vaccination was introduced in the Lazio region, there was encouraging coverage (i.e., 28.5% in 2018 and 43.9% in 2019) and compliance (vaccinated infants received both doses) with HRV (i.e., 87.7% in 2018 and 83.2% in 2019).^52^ Similarly, in the Veneto region, coverage among the 35,393 newborns in 2018 was 84.0% and 81.6% for the first and second dose, respectively, with a compliance rate of 97.1%.^53,54^

The NIP strongly recommends RV vaccination, starting in the third month of life (ages 8–12 weeks). HRV can be administered from 6 weeks of age, according to its SmPC. The interval between first and second dose should be at least 4 weeks, with HRV schedule completion ideally by 16 weeks (maximum 24 weeks of age).^13,55^ According to recently published estimates, delay in completing vaccination and achieving protection could result in around 120 preventable RVGE hospitalizations, costing the health system around €175,000 per year (based on the Lombardy region data).^19^ Additionally, if the HRV schedule is completed within the ideal completion times (16 weeks of age), infants can be protected before they reach the peak age of hospitalization for intussusception (occurring around 16–36 weeks of age).^19^

## Safety

Intussusception is one of the most discussed potential serious adverse events following RV vaccine administration. In Europe, the rate of intussusception without vaccination is about 20–72/100,000 children under 1 year of age, depending on the country.^56^

**Figure 2.**
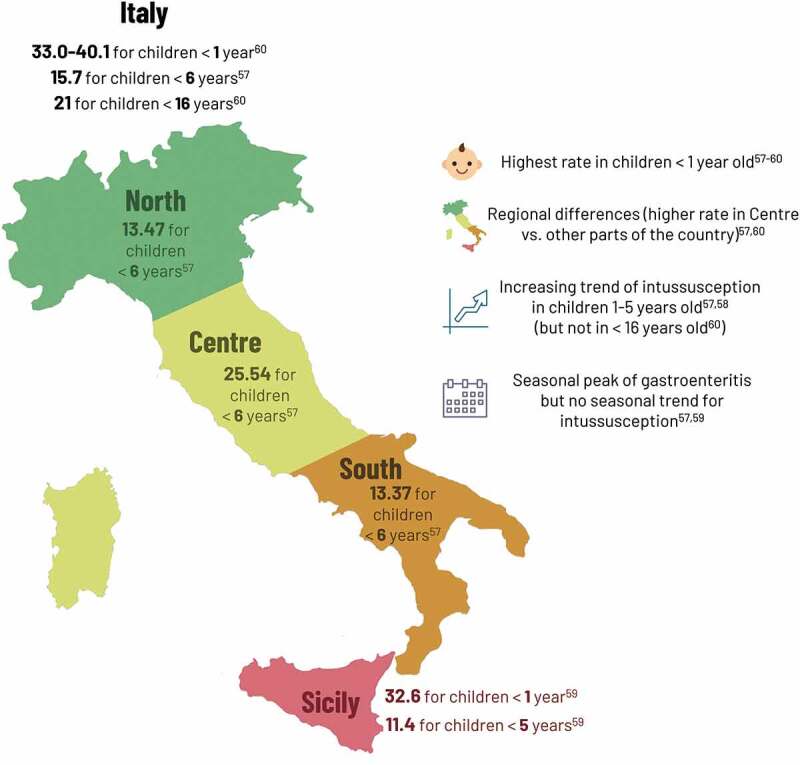
Pre-vaccination intussusception hospitalization rate (/100,000) in Italy.

Several studies assessed the pre-vaccination burden of intussusception in Italy (Figure 2). Higher intussusception hospitalization rates were reported in the Central region^57^ and 7.7% of intussusception cases had previous or concomitant AGE.^58^ Intussusception cases were equally distributed across the year, whereas AGE cases had a seasonal peak in late winter/spring.^5,59^ The risk of intussusception remained highest in children <1 year, however, there was a significant increasing trend of intussusception hospitalizations in some older age groups (1–2 years and 2–6 years) between 2005 and 2014,^57^ with another study reporting increasing incidence rates of 18% overall and 40% in ages 1–5 years.^58^ From the national hospital discharge database, overall incidence in children <16 years of age, however, was stable from 2005 to 2012.^60^

The reported increased post-vaccination risk is low, at around 1–6 additional cases per 100,000, and occurs mostly within 7 days of the first dose.^13,56^ The outcome of studies investigating intussusception post-RV vaccination continue to provide evidence that strongly supports continued vaccination.^56^

In the US, the national passive surveillance system identified a low but significant risk (1.6 excess events per 100,000 vaccinations; 95% confidence interval [CI] 0.3–5.8) of intussusception 3–6 days after dose 1 of HRV. The risk was outweighed by vaccination benefits as there were 68 excess intussusception hospitalizations for 40,000 avoided RVGE hospitalizations per year.^61^

The first study in a European setting (England) that showed a low increased risk of intussusception (1.68 per 100,000 doses) following HRV also found the risk mostly occurred in the first week after the first dose.^62^ A subsequent retrospective ecological study in England for children under 3 years of age, encompassing 20,143,062 person-years, found a post-vaccination increase in intussusception hospital admission rates among infants of vaccination age, which was compensated for by a reduction among older infants. There was no overall change in hospital admission rates or clinical severity of intussusception (i.e., requiring surgery) before and after URV implementation.^63^ The mechanisms following vaccination impacting on intussusception are not known. One hypothesis to explain the increase in risk in young infants is that children who are susceptible may experience intussusception at an earlier age, triggered by vaccination. Another hypothesis, which could explain reductions in overall cases of intussusception in the post-vaccination period, from this and other studies, is that RV vaccination may reduce the risk, as yet unproven, of intussusception from wild-type RV infection in older infants.^63^ However, both hypotheses rely on a direct role of RV on intussusception, which is not sustained by the epidemiologic curve of RVGE and intussusception.^63^

The results of the ecological study carried out in England are in line with a retrospective study carried out at a large pediatric hospital in Florence from 2009 to 2013, which investigated 222 intussusception cases in RV-vaccinated and non-vaccinated children.^64^ Most subjects (84%) presented one time to hospital for intussusception, of which spontaneous resolution occurred in 16% of cases, while 7.4% required an enema, 61% needed hospitalization, and 14.8% underwent surgery. The most frequent symptoms were abdominal pain (41%), vomiting (35%), crying (19%), diarrhea (16%), and blood/mucus in the stool (14%). There were no differences in clinical severity and outcome of intussusception between RV-vaccinated and non-vaccinated children.^64^

The safety of HRV vaccine is further supported by a clinical study in Sicily, where six Neonatal Intensive Care Units enrolled pre-term newborns for RV vaccination. Overall, 449 pre-term newborns, with an average gestational age of 31.4 weeks (standard deviation [SD] 2.7), received HRV at age 6.3 weeks (SD 0.6). Only 8% and 2% of vaccinated newborns reported abdominal colic and fever >38.5°C in the 15 days after the first dose, respectively. No serious adverse events were observed in the 30 days follow up.^65^

Bonanni and Signorelli (2015)^56^ published a report on the serious side effects of RV vaccine, with particular regard to intussusception. This risk was assessed, considering available scientific evidence and other European guidelines, but was not considered an impediment to recommending universal vaccination, as the benefits of vaccination far outweighed the risks.

The Italian National Drug Agency (AIFA) released a safety communication (in 2017) reconfirming the favorable benefit-risk profile of HRV and the positive impact on public health, in line with European-level conclusions, based on review of the evidence including an English study.^62^ However, AIFA recommended that parents must be informed that there is a risk of intussusception within 30 days after RV vaccination, and in the case of specified signs and symptoms (i.e., severe vomiting, diarrhea, blood in feces, abdominal pain, etc.) parents need to seek medical assistance, and the consulted physician would be expected to assess the clinical picture in depth, and the RV vaccine schedule should be completed as per the recommendations and the SmPCs.^62^

In 2017 and 2018, AIFA published an annual report on the vaccine adverse events that were spontaneously reported to the National Surveillance System for Vaccines. In both reports, no deaths were recorded following RV vaccination in Italy, and 2 and 8 cases of intussusception (2017 and 2018) had a possible link to vaccination. The resulting rate of intussusception was 1.5 per 100,000 doses administered, in line with other European countries.^66,67^ In the 2019 report,^68^ when all regions fully implemented RV URV, no specific frequencies were reported for intussusception in the adverse events section for RV vaccines; however, based on reported figures, a rate of 0.6 cases per 100,000 administered doses can be calculated.

Finally, although intussusception, if it occurs in vaccinated babies, is generally referred to RV vaccination, it should be noted that other factors may have contributed to the intussusception event. RVGE itself can be a risk factor to trigger intussusception, as reported in different studies.^69–72^ A case–control study (n = 125 cases and n = 190 controls) of risk factors for intussusception in children aged 0–5 years in Sicily (2009–2015) identified previous AGE (odds ratio [OR] 11.55 [95% CI 3.23–41.23], *p* < .001) and antibiotic use in the 30 days before hospitalization (OR 3.09 [95% CI 1.17–8.12], *p* = .009) as significant risk factors, while infants who had been exclusively breastfed for at least 2 months were at lower risk (OR 0.48 [95% CI 0.23–0.99], *p* = .009). For children born after December 2012, when RV vaccination became available, there was no association between intussusception and vaccination (OR 0.96 [95% CI 0.41–2.25], non-significant *p* = .92).^70^

## Current and future perspectives

Since RV vaccination has been implemented, a number of positive and unexpected findings have been reported, in addition to the effect on RV disease.

Austria was the first European country to implement universal infant RV vaccination in 2007, achieving 87% coverage by 2008. By the end of 2008, hospitalization had already decreased by 74% in the age group eligible for vaccination compared to pre-vaccination rates. In 2009, a further 22% decrease in hospitalization was noted among children aged 32–60 months of age, who were not eligible for the URV program due to their age. The high coverage achieved with URV is expected to have resulted in this indirect herd protection effect, by reducing RV circulation and virus shed from vaccinated children.^73^ Similarly, based on a meta-analysis of studies in children <1 year of age from five countries, the European Center for Disease Prevention and Control reported a median herd effect of 22% on RVGE morbidity (19 − 25%) across 12 study years.^74^ Nevertheless, the mechanism by which the herd effect is generated, for example, through transmission of vaccine virus conferring protection, or through reduced circulation of virus, or reduced numbers of carriers, has not yet been clarified, and further knowledge on RV disease and vaccine effects needs to be accumulated.^74^

Increasing research shows that RV infection is systemic and not confined to the gastrointestinal tract.^75^ Infection can present without diarrhea and may trigger symptoms including neurologic symptoms (e.g., seizures and epilepsy), neonatal complications and autoimmune diseases (e.g., diabetes mellitus and celiac disease), among others. These findings need further investigation, offer new clinical perspectives on RV vaccination, and new opportunities for public health.^76^

Since the implementation of RV vaccination programs, countries have observed reductions in seizure hospitalizations in vaccinated children (e.g., a significant 20% reduction in seizures requiring emergency care or hospitalization in the year following the last RV vaccination).^76^ Seizure hospitalization trends in a US study found the greatest reduction in rates was among children 0–2 months old and 12–23 months old (i.e., a 14–16% reduction in 2013 compared to the pre-vaccination period 2000–2006), and there was a seasonal trend with the largest decreases observed during the RV season.^77^

Similarly, the incidence of type 1 diabetes (T1D) appears to be significantly lower in children who received RV vaccination.^75,78,79^ In children <5 years of age in Australia, there was a 14% reduction in T1D incidence in children born after the introduction of RV vaccination in 2007 (i.e., rate reduction of 0.86 [95% CI, 0.74–0.99], *p* = .04 between 2000–2007 and 2008–2015).)^78^ A cohort study in children in the US compared T1D incidence before (2001–2005) and after (2006–2017) RV vaccination was introduced. Only children who received the full course of vaccination appeared to have a significantly reduced risk of T1D (33% [95% CI 17–46] reduction) and T1D hospitalization (31% [95% CI 27–35] reduction) compared to partially vaccinated and unvaccinated children. The authors conclude that RV vaccination may be “the first practical measure” that could help with the prevention of T1D.^79^

RV vaccination may also help in the prevention of celiac disease – two long-term follow-up studies in Finland found children vaccinated against RV had a lower risk of developing the autoimmune disease than unvaccinated children.^75^

## Conclusions

RV vaccination is highly recommended in Italy. Its benefits have been widely demonstrated in terms of vaccine efficacy and safety, reducing gastroenteritis morbidity, and providing cost savings to the National Health System and society.^1^ This favorable benefit-risk profile and positive experience in Italy to date should support improved coverage and help reach the 95% coverage target.

In addition, high coverage is expected to have generated herd immunity, and new evidence suggests the vaccine may have a positive indirect impact against other diseases such as epilepsy, diabetes and celiac disease.

*Rotarix* introduction in Italy in 2006 was supported by evidence including: clinical trials in Europe, Latin America, Africa, and Asia; epidemiologic burden studies (13 in Italy); healthcare providers opinions (7 studies in Italy); and congress debates. Thus, the value of universal RV vaccination was demonstrated for various stakeholders including payers, healthcare professionals, and parents, which in turn significantly contributed to the implementation of RV universal mass vaccination in Italy, and to the vaccination of more than 1 million babies.

## Supplementary Material

Supplemental Material

## Data Availability

Data sharing is not applicable to this article as no new data were created or analyzed in this research.

## Trademark statement

*Rotashield* is a trademark owned by or licensed to Wyeth Lederle Vaccines SA. *RotaTeq* is a trademark owned by or licensed to Merck & Co, USA. *Rotarix* is a trademark owned by or licensed to the GSK group of companies.
