# Supplementary material for: Fourteen years’ clinical experience and the first million babies protected with human live-attenuated vaccine against rotavirus disease in Italy
Source: Hum Vaccin Immunother. 2021 Aug 9;17(11):4636–45. doi: 10.1080/21645515.2021.1955611 (PMC8828124; doi:10.1080/21645515.2021.1955611)
Supplement: Supplemental Material [file KHVI_A_1955611_SM6013.docx]

Fourteen years’ clinical experience and the first million babies protected with human live-attenuated vaccine against rotavirus disease in Italy

***Journal***: Human Vaccines & Immunotherapeutics

***Authors***

Paolo Bonanni^a^, Giorgio Conforti^b^, Elisabetta Franco^c^, Giovanni Gabutti^d^, Federico Marchetti^e^, Antonella Mattei^f^, Rosa Prato^g^, Giovanni Vitali Rosati^h^, Francesco Vitale^i^

***Affiliations:***

^a^ Department of Health Sciences, University of Florence, Italy

^b^ Italian Federation of Primary Care Paediatricians (FIMP), Genoa, Italy

^c^ Department of Biomedicine and Prevention, University of Rome Tor Vergata, Rome, Italy

^d^ Department of Medical Sciences, University of Ferrara, Via Fossato di Mortara 64B, 44121 Ferrara, Italy

^e^ GSK, Via A. Fleming 2, 37135 Verona, Italy

^f^ Department of Life, Health & Environmental Sciences, University of L’Aquila, Piazzale S. Tommasi 1, 67100, L’Aquila, Italy

^g^ Department of Medical and Surgical Sciences, University of Foggia; Department of Hygiene, Policlinico Riuniti University Hospital of Foggia, Foggia, Italy

^h^ Family Pediatrician (FIMP Federazione Italiana Medici Pediatri), via della montagnola 239, Greve in Chianti (FI), Italy

^i^ Departmentof Health Promotion, Maternal-Child Care, Internal Medicine and Medical Specialties. University of Palermo, Palermo, Italy

***Corresponding author:***

Federico Marchetti

GSK, Via A. Fleming 2, 37135 Verona, Italy

E-mail: federico.e.marchetti@gsk.com

**Table 1.** Hospital and community incidence and burden of rotavirus gastroenteritis in Italy before rotavirus universal routine vaccination.

| Study period  Region |  | Setting  Population | | Incidence | Burden |
| --- | --- | --- | --- | --- | --- |
| **Hospitalized with AGE/RVGE** | | |  |  |  |
| 2003–2012  Sicily  Amodio^1^ |  | • Hospitalized with RVGE  • Children 0–5y | | • N = 9,317 RVGE (62.6% aged 0–23m)  • Rate 3.74/1,000 children/y  • Age 6–11m: 8.85/1,000  • 2003 to 2012: 2.64 to 4.68/1,000 | • Median 4d (IQR 3–6) hospitalization  • Median cost/case €1,536 (IQR 1,279–1,608) |
| 2005–2011  Lombardy, Northern Italy  Pellegrinelli^2^  (Regional HDD) |  | • Hospitalized with AGE  • Children 0–5y | | • N = 32,944 AGE  • RV+: 50.8% (95% CI 50.27–51.35)  • Median RV rate: 0.4% (0.41–0.44)/y | • 65.5% of RV+ were primary diagnoses and most (32.5%) in children aged 12–23m |
| Jan–Dec 2008  Northern Italy  Zuccotti^3^ |  | • Hospitalized RVGE  • Children 0–5y | | • N = 521 AGE  • RV+: 34.9% (95% CI 30.8–39.2)  • 67.6% were aged 0–2y (overall 34.1% were aged 1–2y) | • More severe cases (Vesikari) in RV+ children (69.2% vs 41.4%, *p*<.0001)  • Median 4d hospitalization |
| Winter/spring 2006–2007 and 2007–2008  Italy  Festini^4^ |  | • NRV  • Children 0–30m | | • NRV total incidence (95% CI):  • Overall: 5.3% (3.6–7.5)  • 7.9/1,000d of hospital stay  • >6m vs <6m: 6.3 (4–9.7) vs 4.23 (2.3–7.6) | • Hospital duration sig. increased for NRV+ cases: 8.1d [SD 5.4] vs 6.4d [SD 5.8], *p* = 0.004  • NRV costs around €8.02 million/y |
| 2000–2007  Veneto region  Saia^5^  (Regional HDD) |  | • Hospitalized with RVGE  • Children 0–5y | | • Of 26,202 AGE cases, 54.2% were children 0–5y, of which 28.6% of known etiology were RV+  • RVGE rate: 195.8/100,000  • Age <1y (highest): 271.1/100,000 | • RVGE economic impact: €460,630 to €1,009,600 |
| 2006  Genoa, Northern Italy  Panatto^6^ |  | • Hospitalized with RVGE  • Children 0–5y | | • RV rate: 55/10,000  • Most (29.6%) aged 12–23m | • For community-acquired RVGE, 85.6% had a severe infection (Vesikari score ≥11) |
| Feb 2005–Aug 2006  Five European countries  Forster^7^ |  | • Hospitalized community-acquired AGE  • Children 0–5y | | • In Italy, of 824 AGE cases, 33.0% (29.2–37.0) were RV+, highest in 12–24m (35.4%) | • 80.9% cases in children <2y  • AGE was severe in more RV+ vs RV- cases (Vesikari score ≥11 in 53.3% vs 31.0%) |
| 2001–2005  Four regions in Italy  Marchetti^8^  (Regional HDD) |  | • Hospitalized VE and RVGE  • Children 0–5y | | • RVGE: 2% of all cases and 64.6% of all VE cases  • Age 0–5y RV rate: 279/100,000  • RV primary diagnosis: 158/100,000 |  |
| 2001–2003  Italy  Marocco^9^  (National HDD) |  | • Hospitalized with AGE  • Children 0–5y | | • 20% viral infection, of which 84% were RV+  • RV rate: 177/100,000  • Age <1y RV rate: 255/100,000  • Age 1–4y RV rate: 157/100,000  • May be underestimation as database reported 54% of AGE cases as undefined etiology | • Majority (85%) of RV+ cases in children • 0–5y; 29% in <1y and 71% in ages 1–4y |
| 2000–2003 Emilia Romagna region  Gabutti^10^ |  | • Hospitalized with RVGE  • Children 0–14y | | • 20% viral infection, of which 84% were RV+  • RV rate: 64.7/100,000/y | • RV hospitalization costs the region €400,000–700,000/y |
| Albano^11^ |  | • Hospitalized with AGE  • RV- and RV+ matched children 0–4y | |  | • RV+ cases had sig. longer hospital stays, more severe clinical course (in 15.7% RV+ vs 5.4% RV-, *p*<.0001); sig. longer diarrhea duration, more vomiting, and higher degree of dehydration |
| **Community/primary care +/- hospital** | | |  |  |  |
| Nov 2005–May 2007  Multinational  Diez Domingo^12^ |  | • GP/family pediatrician RVGE visits  • Children 0–5y | | • In Italy, 16.2% were RV+  • Annual rate (95% CI): 13.5/1,000 (10.3–17.4) children-years  • Age 12–24m (highest): 25.8 [16.5–38.6]  • Decreasing in other age groups | • Overall, most (69.1%) were aged <2y and most (92.9%) were rated moderate or severe on the Vesikari scale |
| 2005–2006  Central Italy  Ansaldi^13^ |  | • Community AGE  • Children 0–5y | | • 34.1% of children were RV+  • Age 0–6m: 22.2% RV+  • Age 31–36m: 47.2% RV+  • Age 37–42m: 43.3% RV+  • Cumulative incidence  • Age 0–5y overall: 21%  • Age 7–18m (highest): 41%  • Ages 0–6m: 17.5% |  |
| 2004–2005 season  Seven European countries  Van Damme^14^ |  | • Hospitalized and primary care AGE and RVGE  • Children 0–5y | | • Of 2,846 AGE cases 1,102/2,712 (40.6%) were RVGE+ by ELISA  • RVGE incidence in Italy: 4.70 [95% CI 4.08–5.30]  • Age 6–11m: 8.13  • Age 12–23m: 9.16  • In Italy, 68.8% of hospital cases, 61.4% of ED cases, and 32.9% of primary care cases were RVGE+ |  |
| 25 EU states  Soriano-Gabarro^15^ |  | • Model of RV at home, outpatient, and hospital | | • N = 87,313 RV hospitalizations/y in 25 EU states  • In Italy: 10,053 hospitalizations, 80,425 physician visits, 321,700 home episodes, and 11 deaths/y predicted | • For each hospitalization, there were eight clinic visits, and 32 episodes at home |

95% CI, 95% confidence interval; AGE, acute gastroenteritis; d, day(s); ED, emergency department; ELISA, enzyme-linked immunosorbent assay; EU, European Union; GP, general practitioner; HDD, hospital discharge database; IQR, interquartile range; m, month(s); N, number of cases; NRV (+), nosocomial rotavirus (positive); RV (-/+), rotavirus (negative/positive); RVGE (+), rotavirus gastroenteritis (positive); SD, standard deviation; VE, viral enteritis; y, year(s)

1. Amodio E, Tabacchi G, Cracchiolo M, Sciuto V, Vitale F. Hospitalisation of children aged 0-59 months with rotavirus gastro-enteritis before the introduction of routine vaccination (Sicily 2003-2012). Paediatr Int Child Health 2015; 35:319-323. doi: 10.1080/20469047.2015.1109228

2. Pellegrinelli L, Bubba L, Primache V, Chiaramonte I, Ruggeri FM, Fiore L, Binda S. Burden of pediatrics hospitalizations associated with rotavirus gastroenteritis in Lombardy (Northern Italy) before immunization program. Ann Ist Super Sanita 2015; 51:346-351. doi: 10.4415/ann_15_04_16

3. Zuccotti G, Meneghin F, Dilillo D, Romanò L, Bottone R, Mantegazza C, Giacchino R, Besana R, Ricciardi G, Sterpa A, et al. Epidemiological and clinical features of rotavirus among children younger than 5 years of age hospitalized with acute gastroenteritis in Northern Italy. BMC Infect Dis 2010; 10:218. doi: 10.1186/1471-2334-10-218

4. Festini F, Cocchi P, Mambretti D, Tagliabue B, Carotti M, Ciofi D, Biermann KP, Schiatti R, Ruggeri FM, De Benedictis FM, et al. Nosocomial rotavirus gastroenteritis in pediatric patients: a multi-center prospective cohort study. BMC Infect Dis 2010; 10:235. doi: 10.1186/1471-2334-10-235

5. Saia M, Giliberti A, Callegaro G, Baldovin T, Busana MC, Pietrobon F, Bertoncello C, Baldo V. Hospitalisation for rotavirus gastroenteritis in the paediatric population in the Veneto Region, Italy. BMC Public Health 2010; 10:636. doi: 10.1186/1471-2458-10-636

6. Panatto D, Amicizia D, Giacchino R, Tacchella A, Natalizia AR, Melioli G, Bandettini R, Di Pietro P, Diana MC, Gasparini R. Burden of rotavirus infections in Liguria, Northern Italy: hospitalisations and potential savings by vaccination. Eur J Clin Microbiol Infect Dis 2011; 30:957-964. doi: 10.1007/s10096-011-1180-7

7. Forster J, Guarino A, Parez N, Moraga F, Román E, Mory O, Tozzi AE, de Aguileta AL, Wahn U, Graham C, et al. Hospital-based surveillance to estimate the burden of rotavirus gastroenteritis among European children younger than 5 years of age. Pediatrics 2009; 123:e393-400. doi: 10.1542/peds.2008-2088

8. Marchetti F, Assael B, Gabutti G, Guarino A, Lopalco PL, Marocco A, Ruggeri F, Titone L, Tozzi A, Rosati GV, et al. Monitoring the rate of hospitalization before rotavirus immunization in Italy utilizing ICD9-CM regional databases. Hum Vaccin 2009; 5:172-176. doi: 10.4161/hv.5.3.6764

9. Marocco A, Assael B, Gabutti G, Guarino A, Lopalco PL, Marchetti F, Ruggeri FM, Titone L, Tozzi AE, Vitali Rosati G, et al. [Hospitalisation associated with rotavirus gastroenteritis in Italy, 2001-2003, evaluated by means of ICD9-CM diagnostic codes]. Ig Sanita Pubbl. 2006; 62 (2):215-244.

10. Gabutti G, Lazzara C, Marsella M, Bergamini M, Malaventura C, Borgna-Pignatti C. Burden of hospitalizations due to Rotavirus infection in Emilia Romagna, Italy. Acta Biomed 2007; 78:176-181.

11. Albano F, Bruzzese E, Bella A, Cascio A, Titone L, Arista S, Izzi G, Virdis R, Pecco P, Principi N, et al. Rotavirus and not age determines gastroenteritis severity in children: a hospital-based study. Eur J Pediatr 2007; 166:241-247. doi: 10.1007/s00431-006-0237-6

12. Diez-Domingo J, Baldo JM, Patrzalek M, Pazdiora P, Forster J, Cantarutti L, Pirçon JY, Soriano-Gabarró M, Meyer N. Primary care-based surveillance to estimate the burden of rotavirus gastroenteritis among children aged less than 5 years in six European countries. Eur J Pediatr 2011; 170:213-222. doi: 10.1007/s00431-010-1289-1

13. Ansaldi F, Lai P, Valle L, Riente R, Durando P, Sticchi L, Tucci P, Biasci P, Crovari P, Gasparini R, et al. Burden of rotavirus-associated and non-rotavirus-associated diarrhea among nonhospitalized individuals in central Italy: a 1-year sentinel-based epidemiological and virological surveillance. Clin Infect Dis 2008; 46:e51-55. doi: 10.1086/527449

14. Van Damme P, Giaquinto C, Huet F, Gothefors L, Maxwell M, Van der Wielen M. Multicenter prospective study of the burden of rotavirus acute gastroenteritis in Europe, 2004-2005: the REVEAL study. J Infect Dis 2007; 195 Suppl 1:S4-s16. doi: 10.1086/516714

15. Soriano-Gabarró M, Mrukowicz J, Vesikari T, Verstraeten T. Burden of rotavirus disease in European Union countries. Pediatr Infect Dis J 2006; 25:S7-s11. doi: 10.1097/01.inf.0000197622.98559.01
